# The risk of osteoporotic fractures and its associating risk factors according to the FRAX model in the Iranian patients: a follow-up cohort

**DOI:** 10.1186/s40200-014-0093-2

**Published:** 2014-10-22

**Authors:** Shahnaz Ghafoori, Abbasali Keshtkar, Patricia Khashayar, Mehdi Ebrahimi, Majid Ramezani, Zahra Mohammadi, Farzane Saeidifard, Nasrin Nemati, Maryam Khoshbin, Solmaz Azizian, Fatemeh Zare, Sara Shirazi, Bagher Larijani

**Affiliations:** Endocrinology and Metabolism Research Center, Endocrinology and Metabolism Clinical Sciences Institute, Tehran University of Medical Sciences, Tehran, Iran; Osteoporosis Research Center, Endocrinology and Metabolism Clinical Sciences Institute, Tehran University of Medical Sciences, Tehran, Iran

**Keywords:** Fracture, FRAX, Major osteoporotic fracture

## Abstract

**Background:**

The present study is designed to assess the incidence rate of osteoporotic fracture and its risk factors, particularly those used to predict the 10-year risk of osteoporotic fracture in FRAX based on the data gathered through a follow up cohort initiated in 2000.

**Methods:**

The present retrospective cohort was conducted on men and women from 40 to 90 years of age enrolled in the IROSTEOPs study. A phone survey was conducted during 2013 and beginning of 2014 to assess the fractures (traumatic/osteoporotic) occurring at the time of inclusion until the date of the telephone survey, its type and mechanism, and the patient’s age at the time of accident. Survival analysis using Kaplan-Meier product-limit method was performed with the time of fracture as the study outcome.

**Results:**

Final study population consisted of 1233 individuals, translated in to 9133 person years. The incidence rate of osteoporotic fracture was reported to be 359.1 cases in every 10,000 person years. The 10-year Kaplan-Meier estimate of any kind of major osteoporotic fractures for all the subcohort population was 10.75%. Osteoporosis (HR = 0.75), Discordance between femoral neck and spine (HR = 1.45), Diabetes (HR = 1.81), IBD (HR = 1.84), immobility more than 90 days (HR = 2.19), and personal history of fracture (HR = 7.75) had a considerable effect on the 10-year risk of major osteoporotic fractures.

**Conclusions:**

Adding new clinical risk factors to FRAX® may help improve fracture prediction in the Iranian population.

## Introduction

Osteoporosis is becoming a health concern worldwide, with statistics showing that 200 million adults suffer from the condition [1]. In Iran, 22.2% and 59.9% of women aged over 50 and 11.0% and 50.1% of men of the same age group are diagnosed with osteopenia and osteoporosis, respectively [2]. Recent projections indicate that by 2050, approximately, 44 million will be suffering from some degree of osteopenia and 5 million will be affected by osteoporosis.

Osteoporotic fracture is usually the first manifestation and the main complication of the condition, imposing a heavy burden on the family and society. According to available statistics, 620,000 new hip fractures, 575,000 shoulder fractures, 250,000 proximal humorous fractures and 620,000 symptomatic vertebral fractures were reported in subjects over the age of 50 years in Europe in 2000, representing almost 35% of the fractures reported in the world [3]. The direct costs of osteoporotic fracture in Europe are estimated to be around 36 billion Euros per year [4].

In a study designed to assess falls leading to hip fracture, Abolhassani et al. found that the crude annual incidence of falls and related hip fractures in Iran for those aged over 50 was 237.1 and 93.6 per 100,000 person years, respectively [5]. Based on these results, the number of hip fractures in people aged over 50 years was estimated to be 472.1 cases per 100,000 population in 2010. In a nationwide prospective study designed to assess the burden of hip fracture in Iran, the country accounted for 0.85% of the global burden of hip fracture and 12.4% of the burden of hip fracture in the Middle East [6].

As demonstrated elsewhere, fracture history is one of the strongest risk factors for subsequent fractures [7]. Therefore, it is imperative to identify patients at risk of fracture to implement preventive measures and identify those who would benefit from pharmacological intervention. In response to this demand, recently, a number of prognostic models have been developed which basically rely on the results of bone density measurements as well as some other recognized risk factors for osteoporotic clinical risk fracture (CRFs) [8].

The most widely used osteoporotic fracture risk measurement tool, FRAX®, was invented in 2008 by a WHO Collaborating Center for Metabolic Bone Diseases at Sheffield. The FRAX® tool is developed to compute age-specific fracture probabilities based on CRFs for fracture, identified from large population-based cohorts with over a million person-years of observation, and bone mineral density (BMD) measurements at the femoral neck [9]. The FRAX® tool calculates the 10- year probability of major (clinical spine, hip, forearm or proximal hummers) and hip osteoporotic fracture adjusted to the fracture and death hazards of several countries.

Since its introduction, FRAX® has been calibrated for several nations [10]. In order for FRAX® to be calibrated and used in a particular nation, it is important to design cohort studies by means of which FRAX® parameters can be objectively assessed. Unfortunately the tool is not yet validated for the Iranian population as there has been no population-based cohorts for the development of the FRAX® algorithm in the country.

The extension of the tool for calculating the probability of fractures using the FRAX® is foreseeable in Iran similar to what is occurring in other countries and this would justify a study to allow the necessary adjustments in calibration of the parameters included in the logarithmic formula constituted by FRAX®.

It is therefore reasonable to validate the tool in a cohort on patients who visit different healthcare levels for diagnosis, treatment and follow up of osteoporosis before the generalized use of the tool in medical centers around the country.

Moreover, recent evidence also recommend the evaluation of other risk factors related to low bone mass and higher risk of fragility fracture that are not considered in the FRAX® tool when assessing fracture risk [11,12].

The present study was therefore conducted to assess the incidence rate of osteoporotic fracture and its risk factors and compare them with those used to predict the 10-year risk of osteoporotic fracture in FRAX® based on the data gathered through a follow up cohort initiated in 2000.

## Material and methods

The present retrospective cohort was conducted on men and women from 40 to 90 years of age enrolled in the IROSTEOPs study. The IROSTEOPS (Iranian Osteoporosis Study) is the largest prospective cohort conducted in Iran to predict the prevalence of osteoporosis based on BMD measurement parameters and the recognized risk factors of osteoporosis and related fractures, including those used in the FRAX® tool. This cohort included men and women referred to the BMD clinic of Shariati Teaching Hospital for central bone densitometry by Dual-energy X-ray absorptiometry (DXA) as the initial evaluation of osteoporosis or treatment follow up. During the past decade, more than 13,000 individuals with the median of 1,000 patients per year were visited at this clinic. The referral criteria followed the existing recommendations and was not for screening [13,14]. However considering the fact that our hospital is a referral hospital, it could be concluded that our results is higher than other BMD clinics but more likely to be an estimation of the whole country.

Since the establishment of our BMD clinic, an extensive questionnaire on individuals’ demographic and socioeconomic data, bone-health-related lifestyle habits, medical and drug history, and a semi-quantitative Food Frequency Questionnaires (FFQ) was filled out for every individual. The studied CRFs utilized were those identified from previous meta-analyses. The collected data was stored in a specific database for further studies such as this cohort.

Bone Mineral Density measurements at lumbar spine and hip were determined using dual-energy X-ray absorptiometry (Lunar DPXM, Lunar, 1999) following conventional procedures.

In order to assess the incidence of osteoporotic fracture, a phone survey was conducted during 2013 and beginning of 2014 on those who accepted to answer the follow-up questions. The follow-up study was approved by the Ethical Board Committee of the Endocrinology and Metabolism Research Institute affiliated with Tehran University of Medical Sciences. The telephone questionnaire collected data regarding fractures (traumatic/osteoporotic) occurring at the time of inclusion until the date of the telephone survey, its type and mechanism, and the patient’s age at the time of accident.

Fragility or osteoporotic fractures were defined as those which occur from a fall from a standing height or less, or presenting in the absence of obvious trauma. These fractures were categorized in two main groups: hip fractures or major osteoporotic fractures [15].

Subjects aged less than 40 or over 90 years of age at the time of the first visit to our clinic were excluded since FRAX does not allow the calculation of the adjusted risk outside this age range. Individuals not providing consent to answer the questions as well as those without a telephone to contact, who had moved or their phone number had changed or did not respond after three calls made at different times according to the protocol were excluded.

For the main objective (calculating the incidence rate of osteoporotic fracture rate in a prospective cohort using survival analysis), a sample of 1,000 individuals was needed based on the study conducted by Kanis et al [16].

To minimize the effect of possible losses, which may imply bias (given the mortality associated with fractures and possible address change over 10 years), the sample size was increased to 2,000. This increase also aims to minimize the losses to follow up or refusal to participate. This subgroup constituted 15% of the whole number of patients who had visited our BMD center between 2000 and 2010. In order to minimize the difference between the prevalence of various risk factors in this group compared to the whole population, the subsample was selected through stratified sampling (Each year was considered as a stratification) and the number of samples in each stratification was proportional to size (Table 1).

**Table 1:** **The distribution of the subsample to the whole number of patients visiting the clinic in the past decade**

**First BMD visit**	**SubCohort** **(** **N** **)**	**Total no. of visitors to the BMD clinic**	**Cohort subsample** **/** **total visitors ratio**
2000	381	2506	15.2
2001	217	1595	13.6
2002	382	2785	13.7
2003	216	1303	16.6
2004	97	872	11.1
2005	164	1244	13.2
2006	132	862	15.3
2007	146	880	16.6
2008	164	764	21.5
2009	161	802	20.1
Total	2060	13613	15.1

Descriptive statistics for demographic and baseline characteristics were presented as mean and standard deviation for continuous variables or count (percent) for categorical variables. Simple comparisons of the baseline characteristics were made among the participants and non-participants of the cohort. The Chi-square test was used to evaluate the association between qualitative variables. The Student’s t-test was implemented to evaluate the differences in the distribution of quantitative variables according to the categories defined by a binary exposure.

To assess the differences in the distribution of quantitative variables with more than 2 categories, ANOVA analysis of variance or its corresponding non-parametric test (Kruskal-Wallis) were used. Individuals were grouped into risk quintiles based on the hip and osteoporotic FRAX estimates using BMD, with men and women categorized separately. According to the literature, individuals are classified in four main groups in osteoporosis studies: Men aged less than 50 yrs, Men aged over 50 yrs, Premenopausal Women, and Postmenopausal Women. In view of the fact that women compromised more than 90% of the studied population, all the men were classified in a single group.

The time of the fracture was considered as the study outcome and survival analysis was conducted. The survival curves, for all the study participants and different predictors, were estimated using the Kaplan-Meier product-limit method. The log rank test was used to compare the survival curves between the exposure groups. Univariable and multivariable cox proportional hazard regression were used to determine the hazard ratio of fracture based on risk facture.

The cox model assumption (proportional hazard assumption) was assessed using log-minus-log plot. The cox regression was performed as following: First, potential risk factors, including all indicated in FRAX®, were implemented in the univariable cox regression analysis. Then variables significantly associated with fracture, with p-value lower than 0.20, were retained for the multivariate analysis. The beta coefficient of cox regression indicated hazard ratio with 95% confidence intervals (CIs). Parallel analyses were performed for osteoporotic fractures and hip fractures. All the statistical tests were set two-sided. Significant p-values for multivariable method were set lower than 0.05. Statistical analyses were performed with Stata (Version 11, USA) and SPSS for Windows (Version 16.0, SPSS, Inc., Chicago, IL, USA).

## Results

The final study population consisted of 1233 individuals, translated in to 9133 person years. Mean age at the time of first visit was 54.2 ± 11.5 years in the cohort subsample. The cooperation rate was about 59.9%. One hundred sixty individuals (7.8%) could not be contacted due to address change and 16 (0.7%) had died during the follow-up period.

Osteoporosis at any site was reported in 19.9% of the studied men and 34.8% of the studied women (P-value < 0.001). During the mean follow-up period of 7.41 ± 3.27 years ranging from 5 to 13 years, participants sustained 328 fractures (osteoporotic/traumatic), suggesting an incidence rate of about 359.1 cases in every 10,000 person years. From among them, 23 cases were osteoporotic hip fractures (incidence rate = 25.2 in every 10,000 person years) and 165 were major osteoporotic fractures (incidence rate = 61.3 in every 10,000 person years) (Table 2).

**Table 2:** **The prevalence of CRFs mentioned in FRAX® in different age**-**sex groups**

**Variables**	**Age**-**sex groups**			**Total** **(** **n** **=** **2060** **)**	**P** **-** **value**
	**Men** **(** **n** **=** **166** **)**	**Premenopausal women** **(** **n** **=** **574** **)**	**Postmenopausal women** **(** **n** **=** **1320** **)**		
Corticosteroid use	24/88 (27.3)	50/212 (33.6)	55/511 (10.8)	129/811 (15.9)	<0.001
Type II diabetes	17 (10.2)	25 (6.1)	165 (12.5)	207 (10.1)	<0.001
Parental fracture history	4 (2.4)	25 (6.1)	54 (4.0)	93 (4.5)	0.06
Personal fracture history	10 (6.0)	18 (3.1)	52 (3.9)	80 (3.9)	0.23
History of major osteoporotic fracture	9 (5.4)	11 (1.9)	31 (2.4)	51 (2.5)	0.03
History of rheumatoid arthritis	3 (1.8)	8 (1.4)	5 (0.4)	16 (0.8)	0.02
History of hyperthyroidism	8 (4.8)	32 (5.6)	72 (5.5)	112 (5.4)	0.93
Early menopause (before 45)	-	-	363 (27.5)	363 (17.6)	

**Figure 1:**
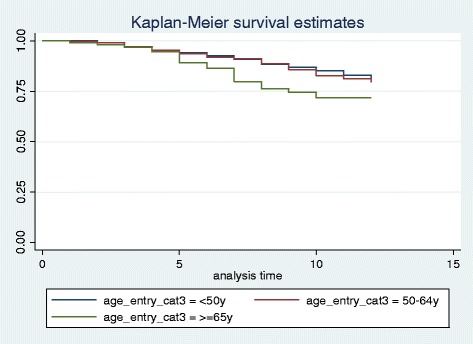
**10-year Kaplan-Meier estimate of major osteoporotic fractures for the subcohort population.**

The 10-year Kaplan-Meier estimate of any kind of major osteoporotic fractures for all the subcohort population was 10.75% (p-value = 0.0046) (Figure 1). For women, the 10-year major osteoporotic fracture risk was 10.45% (p-value = 0.0055).

There was a significant difference between sun exposure among these groups. Based on the results, 76.7%, 78.3%, and 61.4% of postmenopausal women, premenopausal women, and men had no daily sun exposure, respectively. Smoking (p-value = 0.01), and alcohol abuse (p-value = 0.23) were more frequent among men. There was however no significant difference in long-term immobility and walking behavior of the age-sex groups.

The prevalence of corticosteroid use (p-value < 0.001), type II diabetes (p-value < 0.001), rheumatoid arthritis (p-value = 0.02), history of major osteoporotic fracture (p-value = 0.03) were different among the studied groups. While studying demographic variables among those with and without osteoporotic fracture, was the only significantly different variable (Table 3).

**Table 3:** **The demographic variables in individuals with and without a previous history of osteoporotic fracture**

**Variables**		**Without fracture** **(** **n** **=** **905** **)**	**With fracture** **(** **n** **=** **328** **)**	**Total** **(** **n** **=** **1233** **)**	**P** **-** **value**
Gender	Male	52 (5.8)	25 (7.6)	77 (6.2)	0.23
	Female	853 (94.2)	303 (92.4)	1156 (93.8)	
Menopause	Early	143 (15.8)	59 (18.0)	202 (16.4)	0.46
	Normal	444 (49.1)	151 (46.0)	595 (48.3)	
Age at inclusion (Mean ± SD)		54.1 ± 14.5	54.3 ± 11.6	54.2 ± 11.5	0.88
Age at follow-up (Mean ± SD)		62.3 ± 11.8	62.4 ± 12.2	62.3 ± 11.9	0.86
Body Mass Index (BMI)		27.5 ± 0.16	27.5 ± 0.26	27.5 ± 0.14	0.95
Osteoporosis at any site		191/859 (22.2)	84/314 (26.8)	275/1173 (23.4)	0.09
Discordance between lumbar and femoral neck	Major	15/859 (1.8)	16/314 (5.1)	31/1173 (2.6)	0.001
	Minor	257/859 (29.9)	111/314 (35.4)	368/1173 (31.4)	

The resulting osteoporotic fracture incidence rates based on the studied CRFs are listed in Table 4. It could be seen that fracture sufferers were mainly postmenopausal women with a history of early menopause, immobility for more than 90 days, osteoporosis at any site, discordance between spine and femoral neck, rheumatoid arthritis, type II diabetes, hyperthyroidism and IBD. They were more likely to use corticosteroid and to be smoker or use alcohol. We conducted a separate analysis on those who had osteoporotic hip fracture and ended up with the same group of predictors.

**Table 4:** **Incidence rate of osteoporotic fractures based on the studied CRFs**

**Risk factor**	**Classification**	**Osteoporotic fracture** **(** **N** **)**	**At** **-** **risk patient years**	Incidence rate*
Age-sex groups	Men	9	469	191.9
	Premenopausal women	38	2819	134.8
	Postmenopausal women	117	5845	200.2
Menopause age	After 45	84	4479	151.4
	Before 45	33	1366	241.6
Immobility	None	66	3584	184.2
	Less than 90 days	40	1792	223.2
	More than 90 days	20	396	505.1
Osteoporosis femoral neck	Normal	79	5106	154.7
	Osteopenia	59	2907	203.0
	Osteoporosis	19	885	214.7
Discordance between lumbar and femoral neck	None	81	5923	136.8
	Minor	66	2719	242.7
	Major	10	228	438.6
Rheumatoid arthritis	No	155	8689	178.4
	Yes	9	444	202.7
BMI	Normal	51	2735	186.5
	Overweight	70	3951	177.2
	Obese	39	2336	167.0
Type II diabetes	No	139	8372	166.0
	Yes	25	761	328.5
Daily sun exposure	> 30 min	1	152	65.8
	< 30 min	31	1361	227.8
	None	132	7620	173.2
Hyperthyroidism	No	149	8410	177.2
	Yes	15	723	207.5
Corticosteroid use	No	110	6597	166.7
	Yes	54	2536	212.9
IBD	No	134	6867	195.1
	Yes	21	661	317.7
Smoking	No	157	8915	176.1
	Yes	7	218	321.1
Alcohol abuse	No	160	8965	178.5
	Yes	4	161	248.5

*in 10,000 individual.

According to the results of the Multivariate logistic regression analysis, each clinical risk factor had different significance for osteoporotic fracture probability with osteoporosis (HR = 0.75), Discordance between femoral neck and spine (HR = 1.45), Diabetes (HR = 1.81), IBD (HR = 1.84), immobility more than 90 days (HR = 2.19), and personal history of fracture (HR = 7.75) having a considerable effect. The hazard ratios and corresponding 95% CIs associated with each of these variables are presented in Table 5. Hazard ratio estimates with osteoporotic fracture in the whole population were of approximately the same magnitude than those in the female subgroup. This is while age higher than 65 was also associated with 2.62% higher risk of fracture in the whole sub-cohort population.

**Table 5:** **Hazard ratios and corresponding 95% CIs associated with each of the studied CRFs in uni and multivariate analysis**

**Variables**	**Classification**	**Univariate**		**Multivariate**	
		**Crude hazard ratio** **(** **CI95 ** **%)**	**P** **-** **value**	**Crude hazard ratio** **(** **CI95 ** **%)**	**P** **-** **value**
Age-group	<50 yrs	Reference		Reference	
	50-64 yrs	1.07	0.72	0.86 (0.50-1.48)	0.59
	≥ 65 yrs	1.88	0.006	1.77 (0.73-2.58)	0.33
Menopause age	-	Reference		Reference	
	Before 45	1.88	0.008	1.40 (0.77-2.54)	0.27
	After 45	1.43	0.067	1.00 (0.55-1.79)	0.99
T-score at femoral neck	Each SD decrease in T-score	0.68	<0.001	0.75 (0.64-0.89)	0.001*
BMI	Each 1 kg/m2	0.99	0.57	-	-
Type II diabetes	Yes	2.34 (1.52-3.59)	<0.001	1.81 (1.06-3.07)	0.03*
Smoking	Yes	1.78 (0.79-4.03)	0.17	-	-
Corticosteroid use	Yes	1.24 (0.89-1.75)	0.21	-	-
Daily sun exposure	>15 min/day	Reference	-	Reference	-
	None	0.85 (0.53-1.36)	0.49	-	-
Hyperthyroidism	Yes	1.06 (0.60-1.87)	0.84	-	-
IBD	Yes	1.68 (1.05-2.69)	0.03	1.84 (1.13-2.99)	0.015*
Rheumatoid arthritis	Yes	1.13 (0.55-2.29)	0.75	-	-
Discordance between femoral neck and spine	None	Reference	-	Reference	-
	Minor	1.80 (1.29-2.51)	0.001	1.45 (1.00-2.10)	0.05*
	Major				
Parental hip fracture history	Yes	1.08 (0.48-2.45)	0.85	-	-
Personal fracture history	Yes	9.01 (4.85-16.72)	<0.001	7.75 (3.87-15.49)	<0.001*
Immobility	None	Reference	-	Reference	-
	< 90 days	1.18 (0.78 – 1.77)	0.43	1.06 (0.68-1.66)	0.79
	> 90 days	2.70 (1.61-4.51)	0.001	2.19 (1.24-3.86)	0.007*

*CRFs with significant effect on fracture risk.

## Discussion

Several studies showed that the presence of the risk factors used to trigger a BMD test is associated with a fracture risk greater than that can be accounted for by BMD alone [17,18]. Thus, the assessment of fracture risk should take account of specific risk factors that contribute to fracture risk in addition to BMD, since this would increase the sensitivity of fracture prediction.

WHO fracture risk assessment tool is a scale including 11 of the CRFs (age, body mass index (BMI), prior fragility fracture, parental history of hip fracture, current tobacco smoking, long-term use of oral glucocorticoids, rheumatoid arthritis, other causes of secondary osteoporosis, daily alcohol consumption), which have demonstrated a strong association with the incidence of fracture in literature [19]. Factor number 12 in this scale also includes a single value of DXA: the T-score of the femoral neck.

The relationships between risk factors and fracture risk incorporated within FRAX® have been constructed based on information derived from nine population-based cohorts from around the world and has been validated in 11 independent cohorts (mainly women) with a similar geographic distribution with in excess of 1 million patient years [20]. The large sample used for the model construction permits the determination of the predictive importance in a multivariable context of each of the risk factors, as well as interactions between risk factors, and thereby optimizing the accuracy by which fracture probability can be computed.

However, it is demonstrated that fracture risk assessment cannot entirely replace objective examinations, as the 10-year probability of fracture varies markedly in different countries [21,22]. Moreover, several studies have reported that the number of cases of fracture estimated or expected by FRAX® are significantly lower than the incidental fractures actually observed in the 10 years of follow up [23]. According to the French OFELY cohort, the predicted major osteoporotic fracture probability among women aged 65 years and older was 48% lower than the actual observed fracture incidence [24]. Moreover, a recent study from New Zealand in older postmenopausal women also showed that FRAX® underestimated osteoporotic fractures [25]. However, in a preliminary report from the Framingham Osteoporosis Study, FRAX® yielded excellent results in predicting the probability of hip fractures [26]. This inconsistency may be due to differences not only in risk factor distribution but also in the definition of osteoporotic fracture.

Furthermore, others have pointed out the importance of other risk factors such as vitamin D deficiency, falls, physical activity, bone turnover markers, previous treatment for osteoporosis, and certain medications for decision-making in the management of osteoporosis in primary healthcare settings [27]. The measurement of a risk factor for diagnostic use, however, can only capture one aspect of the likelihood of the outcome when the disease is multifactorial such as osteoporosis.

Although it can be argued that the smaller proportion of men in the studied populations could have posed some limitations to the results, there was reasonable agreement between estimated 10-year Kaplan-Meier estimate of osteoporotic fractures between women and the whole subcohort population.

The prevalence of fracture obtained in the current study was 359.1 in every 10,000 person year (equal to 10.75%). This rate (191.9 in men and 134.8 in premenopausal and 200.2 in postmenopausal women) is higher than the prevalence reported elsewhere in the literature [28]. The ECOSAP cohort showed the incidence of hip and major osteoporotic fracture in the Spanish population to be about 0.96% and 3.81% [29]. In one study on the Canadian population, Leslie et al. reported the incidence of osteoporotic fracture to be about 10.7% in men and 12% in women [30]. Maharlouei et al. reported the incidence rate of hip fracture in men and women from Shiraz was 329 and 580 cases in every 100,000 population [31]. Valizadeh et al. similarly reported the incidence of hip fracture in Zanjan to be 206.5 and 214.8 per 100,000 men and women, respectively [32].

Another problem concerns the threshold for management application. In other words, the scale should be developed and validated in each country, taking into account the differences in fracture and risk factor epidemiological data, their mortality data as well as cost-effectiveness studies to obtain an approximation of the cost, which each country is willing to accept as reasonable for the prevention of fragility fractures.

Thus the FRAX® models need to be calibrated to those countries where the epidemiology of fracture and death is known. As a result, in order to analyze the association between clinical and environmental risk factors (those mentioned in FRAX®) and the occurrence of osteoporotic fracture in the Iranian population, a clinical cohort was designed to promote the study of different risk factors of presenting osteoporotic fractures. The current study is the first such study in the Iranian population.

In corroboration with previous studies, our findings demonstrated that fracture risk increased with increasing age and decreasing T-score. Hui et al. reported that the annual incidence of hip fracture increased by approximately 30-fold between the ages of 50 and 90 years [33]. Similarly, Kanis et al. showed that the 10-year hip fracture probability increases from 2% in women aged 50 to 12% in women aged 80 years for the same T-score [34]. In the present study, in consistency with the aforementioned findings, each year increase in age after the age of 65 years was associated with 2.62% higher risk in the whole subcohort population.

Several prospective studies have indicated that the risk of fracture about doubles for each SD reduction in BMD, which is much higher than that reported in the present study [35]. This could be due to the fact that the majority of those studies were conducted on postmenopausal women, whereas women with a broader age range were included in our study.

Similarly, prior osteoporotic fracture is known as a strong predictor of subsequent fracture. While Pluskiewicz et al. showed that the presence of prior fracture increased by 1.76 -fold for any osteoporotic fracture, the multivariable analysis in our study reported a 7.75-fold higher risk in this regard [36].

Our study revealed that immobility for more than 90 days is associated with a higher risk of major osteoporotic fracture. Melton et al. similarly showed that immobility for more than 90 days doubles the risk of hip fracture [37].

Diabetes is another important risk factor for osteoporotic fracture. In corroboration with previous studies, our results showed that diabetes increases the risk of osteoporotic fracture by 1.8 [38].

Similar to previous studies, our results reported that glucocorticoid use is associated with a higher risk of fracture, the association however was not significant [39]. The reasons for this discrepancy are not fully understood. It might be related to differences in the study populations, especially with regard to age and the type of incident fractures or the low power of the study regarding glucocorticoid use.

Unlike previous studies, BMI in our study did not have a marked effect on fracture probability [40]. This could be due to the fact that BMI could be influenced by height loss associated with vertebral fractures and deformities. Davidson et al. similarly reported that the risk conferred through BMI could be under- estimated in individuals with significant height loss [41].

In several studies, falls were reported to be the most important clinical risk factors for fractures [42,43]. In the present study, however, history of falls was not identified as a strong factor, mainly because not many participants reported such incidents.

This retrospective cohort revealed that several CRFs included in FRAX were not significantly associated with the risk of fracture in our population. Specifically speaking, osteoporosis, diabetes, IBD, immobility for more than 90 days and personal history of fracture were the only significant and independent predictors of osteoporotic fracture. This may be mainly interpreted as the lack of predictive value of these CRFs in the target population, which is the main difference between our cohort and those used to develop the FRAX® tool. Lack of statistical power particularly regarding history of smoking, alcohol use, and other infrequent or under-reported CRFs might also explain the difference.

Apart from the risk factors taken into account in the FRAX® score calculation, discordance between spine and femoral neck BMD was a significant predictor of osteoporotic fracture. It can be proposed that adding new parameters to FRAX® may help improve fracture prediction value of the FRAX tool. Further studies are needed to develop a more predictive model in our population.

### Limitations

Since the cohort is constituted of subjects requiring a DXA scan (according to their physician), it is likely that the recruited population will be at a baseline risk greater than that of the general population. Our results may therefore be extrapolated to a population in which the physician is evaluating the risk of low bone mass or fracture (case finding) which is to some extent similar to the population recommended for investigation by the WHO.

There may be a bias in the collection of the information on incidental fractures, which is collected based on the patient self-report as we did not include radiological assessment since the patients included were from the general community and were not seeking medical attention for bone-related conditions. Moreover, the majority of vertebral fractures are not clinically diagnosed [44]. Moreover, recall is subject to errors. Additionally smoking and alcohol abuse are underreported as the validity of self-reported alcohol intake is notoriously unreliable [45]. The other possible limitation inherent to data collection by telephone was minimized with trained interviewer. However, the reported associations may actually be stronger than reported here.

Our study had also several strengths. Unlike many previous studies that were conducted on postmenopausal women, we enrolled a large group of patients of both genders and the age range was fairly broad. Also the large number of studied risk factors allowed obtaining reliable fracture risk assessment.

In view of the fact that hip fractures represent the minority of osteoporotic fractures [29] focus on hip fractures alone could be misleading for high-risk younger individuals whose 10-year risk relates more to spine and wrist fractures. This is the reason for which not only in FRAX® but also in our study the patient’s 10-year likelihood of any one of four major osteoporotic fractures are calculated.

## Conclusions

In conclusion, only a limited number of CRFs were found to be associated with the higher risk of major osteoporotic fracture. In this population, the majority of risk factors mentioned in the FRAX® tool along with others such as discordance between lumbar spine and femoral neck were associated with increased risk of both hip and major osteoporotic fractures. These considerations indicate that assessment can be improved by the integration of other CRFs into the model. Further studies are needed to determine a model to improve the sensitivity and specificity of clinical scores to identify those at high risk of fracture.
